# Endophytic *Aspergillus fijiensis* fungus J7 prevents disease and promotes growth in *Salvia miltiorrhiza*

**DOI:** 10.1128/spectrum.03094-24

**Published:** 2025-07-30

**Authors:** Xiangyi Yang, Xinyi Cheng, Ziyi Zhao, Mingkun Ai, Hui Liu, Yan Zhu, Feng Han, Minjian Qin, Guoyong Xie

**Affiliations:** 1 Department of Resources Science of Traditional Chinese Medicines, School of Traditional Chinese Pharmacy, China Pharmaceutical University 540414 , Nanjing, China; 2 Yangzhou Center for Food and Drug Control, Yangzhou, China; 3 Chongqing Institute of Medicinal Plant Cultivation, Chongqing, China; 4 Medical Botanical Garden, China Pharmaceutical University 56651, Nanjing, China; Universita degli Studi del Molise, Campobasso, Campobasso, Italy

**Keywords:** *Aspergillus fijiensis*, biocontrol, growth-promoting, *Salvia miltiorrhiza*, metabolic regulation

## Abstract

The objective of this study was to evaluate the feasibility of using the endophytic fungus J7 as a biocontrol agent for *Salvia miltiorrhiza* blight, and to provide ideas for sources and practical applications of biocontrol agents. *Aspergillus* sp. J7 was isolated from *Solidago canadensis* L. and identified as *Aspergillus fijiensis*. The growth inhibition capacity of J7 against *Phytophthora cactorum*, the cause of blight in *S. miltiorrhiza,* was 90.0% in filtrate-modified plates, and the incidence of *S. miltiorrhiza* blight in potted plants was reduced by 14.8%, surpassing the 7.4% reduction in the positive control group treated with cymoxanil plus mancozeb. Strain J7 also significantly increased the fresh weight and height of potted *S. miltiorrhiza* seedlings by 2.39 and 1.37 times, respectively. Furthermore, within 5 months of inoculation, the tanshinone content in the field-grown *S. miltiorrhiza* increased significantly compared to control plants. Additionally, the mechanisms of growth promotion, antimicrobial activity, and regulation of secondary metabolites by strain J7 were explored, which strongly support the practical application of this fungal preparation in controlling *S. miltiorrhiza* blight.

**IMPORTANCE:**

*Phytophthora cactorum* can cause various plant diseases, and currently available control methods are still limited. Existing *Aspergillus* species show certain limitations in the control of plant diseases caused by *Phytophthora*. This study confirms a promising biological control agent that can be used to control *P. cactorum* and provides additional information on the biological characteristics of *Aspergillus fijiensis*.

## INTRODUCTION

The genus *Aspergillus* is a widely distributed fungus commonly used in food, pharmaceuticals, cosmetics, and biocontrol agents. It is an important industrial source of enzymes and metabolites, significantly impacting the economy and society (1–3). It is well known that they can cause food spoilage and synthesize mycotoxins, and they are frequently reported as pathogens in humans and plants (4–6). On the other hand, they can also be used in the fermentation industry to produce hydrolytic enzymes and organic acids (7), and they have recently emerged as a new multifunctional biocontrol agent (8). In most reports concerning the biocontrol of *Aspergillus* strains, *Aspergillus* biocontrol strains are used to manage mycotoxin-producing strains (9, 10). Currently, the ability of *Aspergillus* biocontrol strains to eliminate aflatoxins has been widely assessed, but their potential use in controlling fungal pathogens is still under investigation. Research has reported the use of *Aspergillus flavipes*, *Aspergillus fumigatus*, *Aspergillus japonicus*, and *Aspergillus westerdijkiae* strains to control plant pathogens (11–13). In summary, *Aspergillus* is a promising alternative for controlling fungal and oomycete pathogens, but it shows deficiencies in controlling pathogenic strains such as *Phytophthora* (14). Therefore, the exploration of new *Aspergillus* strains that complement the biology of *Aspergillus* strains and provide effective and safe biocontrol alternatives against fungal and oomycete pathogens is an interesting research endeavour.

Strain J7 was isolated as an endophytic fungus, and endophytic fungi have extensive interactions with their plant hosts, playing important roles in various life activities of plants (15). It has been reported that endophytic fungi can promote plant growth by producing metabolites such as growth hormones and by enhancing processes including nitrogen fixation and phosphorus solubilization (16, 17). They can also enhance the host plant’s tolerance to biotic and abiotic stresses through mechanisms such as competition for space or nutrients, parasitism, and the production of secondary metabolites (18–20). Currently, screening of biocontrol candidate strains is conducted through simple, reliable, and single-variable *in vitro* experimental methods. The biocontrol efficacy of the strains is ultimately verified through tripartite interactions among biocontrol candidate, phytopathogen, and plant host, which is an effective approach for developing biocontrol agents from endophytic fungi (21).

In this study, *Aspergillus* sp. J7 was isolated from the stem of *Solidago canadensis*. The species of the genus *Aspergillus* are found in various habitats around the world, and the currently recognized species list of this genus includes 339 species, making it one of the most difficult groups to classify. Due to the challenges of identifying *Aspergillus* based solely on morphological characteristics, new molecular methods, including sequence markers such as internal transcribed spacer (ITS), calmodulin, *β*-tubulin, translation elongation factor-1 alpha (*TEF-1α*), and RNA polymerase II (*RPB2*), have provided more accurate methods for the taxonomic identification of *Aspergillus* fungi (22, 23). We used a polyphasic approach to identify J7 as *Aspergillus fijiensis*. This is the first report of this species for use in biocontrol. *A. fijiensis* is a less commonly recorded species within the *Aspergillus* group and is a uniseriate species related to *Aspergillus aculeatinus*, first isolated from soil in Fiji and from *Lactuca sativa* in Indonesia. Through secondary metabolite profiling, this strain was found to produce asparagine and okaramins (24). We evaluated *A. fijiensis* J7 as a biocontrol agent by exploring its antifungal and growth-promoting mechanisms, comparing the antifungal efficacy of co-culturing the strain with other strains and using the *Aspergillus* culture supernatant, and analyzing the efficacy of *Aspergillus* spores against pathogenic strains in pot experiments, as well as their growth-promoting effects in field trials.

## MATERIALS AND METHODS

### Phylogenetic analysis and morphological identification of strain J7

J7 was isolated from *S. canadensis* from the Garden of Medicinal Botany, China Pharmaceutical University, Nanjing, Jiangsu Province, China (118″54′E, 31″54′N). Strain J7 was deposited at the Agricultural Culture Collection of China (ACCC) (accession number ACCC 35459).

For mycelium production, a suspension of spores from J7 was grown in potato dextrose agar (PDA) medium (glucose 20 g, potato 200 g, agar 18 g, and distilled water to 1,000 mL, pH 6.5). Mycelia were filtered and lyophilized for total DNA isolation. The fungal DNA was extracted with mechanical grinding using 5 mm iron beads in a Freezer Mill JXFSTPRP-COBL, and a Fungi Genomic DNA Extraction Kit, starting from 10 mg of lyophilized mycelium. The quality of genomic DNA was determined by electrophoresis and it was quantified using an ND-1000 (NanoDrop) spectrophotometer. Calmodulin (CaM, *ca*. 650 nt) was amplified using CL1 and CL2A primers (25), the nuc rDNA ITS rDNA region (*ca*. 645 nt) was amplified using ITS1/ITS4 primers (26), *TEF-1a* (*ca*. 700 nt) was amplified using A-TEF_F/A-TEF_R primers (27) and *RPB2* (*ca*. 950 nt) was amplified using primers 5F and 7CR (28).

Using MEGA 7 software, multiple sequence alignments were performed for each gene, followed by manual trimming. Then, the Sequence Matrix software was used to concatenate the four genes into a single file. Model parameters were calculated using jModeltest 2.1.10, resulting in the best-fit model being TIM3+G. A Markov Chain Monte Carlo algorithm was used to generate phylogenetic trees with Bayesian probabilities using MrBayes v3.2.7 for the combined sequences data set, running for 2,000,000 generations with sampling every 1,000 generations, resulting in 2,000 trees, of which 25% were discarded, and the remaining trees were used to calculate posterior probabilities. The Maximum Parsimony phylogenetic tree was constructed using PAUP 4.0b10 software, utilizing the heuristic search option and the Close Neighbor-Interchange algorithm. The initial tree was obtained by randomly adding sequences, followed by 1,000 bootstrap validations.

Strain J7 was inoculated on four types of culture media, namely Czapek agar (CA), Czapek yeast extract agar (CYA), Malt extract agar (MEA), and 25% glycerol nitrate agar (G25N) (Online Resource Table S1), and cultured in the dark at 25°C for 7 days before the morphological characteristics of strain J7 were observed and recorded.

### Antifungal activity test of strain J7 and investigating inhibition mechanisms

To test the control of J7 against phytopathogenic fungi, 10 common plant pathogens were selected for the experiment. *Phytophthora cactorum* (ACCC 39131) and *Fusarium oxysporum* (ACCC 39331) were purchased from the ACCC. *Fusarium solani*, *Fusarium acutatum*, *Botrytis californica*, *Arthrinium arundinis*, *Fusarium falciforme*, *Epicoccum sorghinum*, S*clerotium rolfsii* Sacc, and *Athelia rolfsii* were obtained from the Chongqing Institute of Medicinal Plant Cultivation. Fungi were cultivated in a dark environment on PDA medium at 25°C for subsequent studies. Strains were inoculated in PDA medium (90 mm) and incubated at 25°C in the dark for 7 days for activation. A 6 mm diameter fungal plug of J7 was obtained using a perforator and placed at both ends of the medium (1.5 cm from the edge), and a pathogenic fungal plug of the same size was placed in the middle of the medium for the co-cultivation test. A blank control was set up, wherein only the pathogenic plug was placed in the medium, and incubation was conducted for 7–10 days until the medium of the blank control was full of fungal mycelia (29). Inhibition was expressed as the mean of triplicates for each sample tested. The inhibition rate was calculated as follows: *n* = [(*A*−*B*)/*A*] × 100%, where *A* is the diameter of uninhibited fungal colonies and *B* is the diameter of co-cultured fungal colonies.

Secondary screening for inhibitory activity was performed using the filtrate-modified medium. Strain J7 was cultured on PDA for 7–10 days until spores were formed and a spore suspension was obtained. An aliquot (1 mL) of spore suspension was added to potato dextrose broth (PDB) and incubated at 25°C for 7 days, then filtered to obtain a sterile culture filtrate. The sterile filtrate was added to PDA medium at a ratio of 1:5 of the total volume to prepare the filtrate-modified medium. The 0.05% (vol/vol) Tween-80 sterile water was added to PDA medium at a ratio of 1:5 of the total volume to create a blank control medium (30). The pathogenic oomycete/fungal plugs were inoculated in the center of the modified medium and blank control medium. The inhibition rate was calculated as follows: *n* = [(*A*−*B*)/*A*] × 100%, where *A* is the diameter of fungal colonies on blank medium and *B* is the diameter of fungal colonies on modified medium.

Biocontrol results either from competition for nutrients and space or from the ability to resist metabolites. Cellulose is abundant in oomycetes, and proteases are highly effective in inhibiting them (31). Assessing the ability of J7 to secrete three cell wall-degrading enzymes (casein protein, cellulase, and *β*-glucan) was a preliminary investigation into the mechanism by which J7 inhibits fungal growth. The casein protein, cellulase, and *β*-glucan medium (Online Resource Table S1) were used in the assay to determine the extracellular enzymes secreted by strain J7. Fungal plugs were inoculated into the assay medium and incubated at 25°C for 2–5 days to observe any color reaction (32), and three technical replicates were performed for each assay.

### Evaluating the biocontrol effect of strain J7 against *P. cactorum*-induced *S. miltiorrhiza* blight in pots

To observe the tripartite interactions among the biocontrol strains, plant pathogens, and plant hosts, the plant pathogen (*P. cactorum*) that was most effectively suppressed was selected for subsequent experiments. Surface-sterilized *S. miltiorrhiza* seeds were sown on the surface of an autoclaved soil mixture consisting of nutrient soil and field soil in a 1:1 (wt/wt) ratio (pH: 5.5–7.5, nitrogen, phosphorus, and potassium content ≥2%, and organic matter ≥50%). The seedlings were incubated in a greenhouse at 25°C under a light cycle of 16 h of light and 8 h of darkness, with a light intensity of 4,000 Lx, and average relative humidity of 70%. Once the seedlings reached the four- to six-leaf stage, they underwent three treatments: Negative Control: 3 mL of 0.05% Tween-80 by root irrigation. Positive Control: 3 mL of 72% cymoxanil plus mancozeb (500 × dilution with 0.05% Tween-80) by root irrigation. Experimental Group: 3 mL of strain J7 spore suspension (1 × 10^6^ spores/mL) by root irrigation. Three days after pretreatment, each pot received 1 mL of *P. cactorum* mycelium (10 mg/mL) by root irrigation. The *P. cactorum* mycelium was obtained by shaking it in PDB medium for 7 days (180 r/min at 25°C). Each group consists of 27 plants, divided into 9 independent replicates, with 3 plants per replicate. Seven days post-inoculation, the incidence, disease index (DI), and control effects (CEs) were recorded. Disease severity was categorized into six classes based on the leaf area affected: 0, 1, 3, 5, 7, and 9 (0%, <10%, 10–25%, 25–50%, 50–75%, and >75% of total leaf area, respectively). DI (%) = ∑ [(number of diseased plants in each class × class)/(total number of investigated plants × highest level value)] × 100.

### Evaluation of the growth-promoting effect of strain J7 on *S. miltiorrhiza* seedlings in pots

Surface-sterilized *S. miltiorrhiza* seeds were sown in pots under uniform soil and greenhouse conditions until the seedlings developed two true leaves. The experimental group received root irrigation with 100 µL of J7 solution (1 × 10^6^ spores/mL containing 0.05% Tween-80) every 5 days, while the control group was irrigated with the same volume of sterile 0.05% Tween-80 solution. Every 10 days, 15 plants were randomly sampled from each group and organized into 5 independent biological replicates per group, with each biological replicate containing 3 plants. Data on plant height, root length, and fresh weight were recorded, with three technical replicates measured per plant and averaged for analysis.

In natural environments, iron is predominantly in the insoluble form Fe³^+^, which severely limits its bioavailability to plants. However, siderophore-producing fungi enhance plant iron acquisition through chelation-mediated solubilization, representing a crucial mechanism for fungal-mediated plant growth promotion (33). Microbially derived growth-promoting hormones (e.g., indole-3-acetic acid, IAA) translocate to plant tissues, where they positively regulate developmental processes through phytohormonal signaling pathways (17). To analyze the growth-promoting mechanism of strain J7 in seedlings, the production of siderophore was evaluated in chrome azurol S (CAS)-PDA medium (Online Resource Table S1). The ability of strain J7 to produce IAA was assessed using Sackowski’s reagent after incubation in PDB with 2.5 g/L tryptophan for 7 days with shaking. The phosphorus-solubilizing ability of strain J7 was evaluated in the medium containing insoluble inorganic phosphorus. The optical density of the phosphate standard solution was measured at 730 nm using a spectrophotometer, and a standard curve was generated (Online Resource Fig. S1). A 1 mL suspension of J7 spores (1 × 10^6^ spores/mL) was added to a medium (Online Resource Table S1) containing Ca_3_(PO_4_)_2_ (10 mg/mL), FePO_4_ (5 mg/mL), and AlPO_4_ (5 mg/mL), then incubated on a shaker at 25°C and 180 rpm. Supernatant samples (1 mL) were collected on days 2, 4, 6, 8, and 10 to measure dissolved phosphorus by molybdenum-antimony colorimetry (34).

### Inoculating J7 on *S. miltiorrhiza* in the field

We selected healthy and uniformly sized annual medicinal *S. miltiorrhiza* plants purchased from Linyi City, Shandong Province, China. The above-ground parts were removed with scissors, and the fibrous roots were trimmed, leaving approximately 15 cm of the main roots. The plants were then weighed and numbered using an electronic balance. Two experimental plots, each measuring 600 × 300 cm, were established in the Garden of Medicinal Botany at China Pharmaceutical University in Nanjing. Nanjing is situated in the northern subtropical monsoon climate zone. During December (the planting season in this study), the region experiences typical winter conditions, with an average monthly temperature of 3.9°C and minimum temperatures reaching −5.8°C. According to data from the Nanjing National Benchmark Climate Station, the cumulative precipitation was 11.2 mm with 170.2 total sunshine hours during this period. One hundred *S. miltiorrhiza* plants were planted in each plot. The field was irrigated with tap water every 2 days, and after the plants developed new above-ground parts, 100 plants in one experimental field were irrigated with 10 mL of the J7 spore suspension (1 × 10^6^ spores/mL), designated as the treatment group. Plants in the other field received the same volume of a blank solution and served as the control group. Treatments were applied at 1-month intervals, and after 3 months, samples were collected from each experimental field using a 5-point sampling method for weight comparison and content determination.

### Determination of content and change in fresh weight

Five independent biological replicates, each containing three *S*. *miltiorrhiza* plants, were collected from the treatment and control groups, and three technical replicates were measured for each plant. Samples were washed to remove soil, and their fresh weights were measured. Fresh weight gain was calculated as the mean ± SD of the fresh weight at sampling minus the initial weight recorded in December.

*Salvia miltiorrhiza* contains three major lipophilic tanshinone derivatives: dihydrotanshinone I, cryptotanshinone, and tanshinone IIA, all of which have well-characterized bioactivities. Pharmacopoeial standards establish tanshinone IIA as a quality marker, with the *Chinese Pharmacopoeia* (2020 edition) specifying its quantification requirements and the *European Pharmacopoeia* mandating a minimum content of 0.12% (wt/wt) in dried medicinal material. Dihydrotanshinone I is considered to be one of the major antitumor components of *S. miltiorrhiza* (35), and further studies confirmed that cryptotanshinone and dihydrotanshinone I had the strongest antimicrobial activity *in vitro* among all tanshinones (36). Therefore, in this study, dihydrotanshinone I, cryptotanshinone and tanshinone IIA were selected as quantitative markers. Samples were dried at 50°C until a constant weight was achieved, then the dried samples were analyzed for changes in tanshinone content using HPLC. Each sample was crushed using a mortar and pestle, precisely weighed, and treated with 50 mL of methanol per 0.3 g of sample. The mixture was sonicated for 30 min (100 kHz) at 25°C, then re-weighed after cooling. Any weight loss was compensated by adding methanol. The concentrations of the three tanshinones in the samples were expressed as μg/g and analyzed via linear fitting using Origin 2021. Chromatographic parameters and methodological investigations are available in the Supplemental material.

### Evaluating the colonization ability of strain J7 in the roots of *S. miltiorrhiza* seedlings

To assess the ability of J7 to colonize *S. miltiorrhiza* in soil, the pCAMBIA 1303-gpda-hyg-gfp plasmid was integrated into J7 through *Agrobacterium tumefaciens-*mediated transformation (37). Specific primers for the *GFP* fragment were designed for quantification using real-time qPCR (QGFP-F: 5′-TTCTTCAAGGACGACGGGGAA-3′, QGFP-R: 5′-AAGTTGGGCTTTGATGCCGTT-3′). Fungal biomass was quantified in *S. miltiorrhiza* plants inoculated with strain J7, grown in sterilized soil and in the field, by extracting total gDNA from the roots at 3, 5, 7, and 15 days post-irrigation with a spore suspension of the transformant strain J7 (1 × 10^6^ spores/mL). Relative quantities of fungal genomic DNA were calculated from the standard curve, and this curve was created by plotting the logarithm of known DNA copies (10-fold dilution series from 10 pg to 10^−4^ pg/10 µL reaction) of the plasmid against the Ct values. Real-time qPCR data are presented as the mean ± SEM of five independent biological replicates. Each biological replicate consisted of three plants, with three technical replicates performed per sample.

### Effect of strain J7 on the expression of tanshinone-related genes in *S. miltiorrhiz* roots and rhizomes

Seedlings at the four- to six-leaf stage were stressed with two treatments: (i) 3 mL spore suspension (1 × 10^6^ spores/mL) of strain J7 with 0.05% Tween-80, (ii) 3 mL 0.05% Tween-80 sterile aqueous solution. *S. miltiorrhiza* seedlings were collected at 0, 3, 6, 12, 24, 36, and 72 h after treatment and analysed by qRT-PCR after snap freezing in liquid nitrogen and grinding.

Real-time qPCR reactions were performed according to the instructions of the dye-based fluorescence qPCR assay kit ChamQ SYBR qPCR Master Mix (High ROX Premixed) (Q341-02, Nanjing Novozymes Bioscience and Technology), with the selection of key enzyme genes for tanshinone-related synthesis, *HMGR*, *DXS*, *DXR*, *GGPPS*, *CPS1*, *CPS5*, *KSL*, and *cyp76AH1* (38). The qPCR amplification conditions: 95°C 30 s, cycle 1 time; 95°C 10 s, 60°C 30 s, cycle 40 times; Annealing: 95°C 15 s, 60°C 60 s, 95°C 15 s. The relative expression of target genes was calculated using the 2^−ΔΔC*t*^ method, with *β*-actin expression as the internal reference. Each experimental group contained three independent biological replicates. For each biological replicate, tissues from three plants were homogenized together, and the pooled sample was subjected to three technical replicate measurements.

### Data analysis

Data were checked for homogeneity and analyzed by one-way ANOVA followed by the Student-Newman-Keuls multiple comparison test or the independent-samples t-test with SPSS 20.0 software (IBM, Armonk, NY, USA). A significance level of *P* < 0.05 was used. Error bars represent structural entropy measure (SEM), and data in tables are presented as the mean ± SD. Graphics were compiled using Origin 2021 (OriginLab, Northampton, MA, USA) software.

## RESULTS

### Morphological and phylogenetic analysis of the identification results

**Fig 1:**
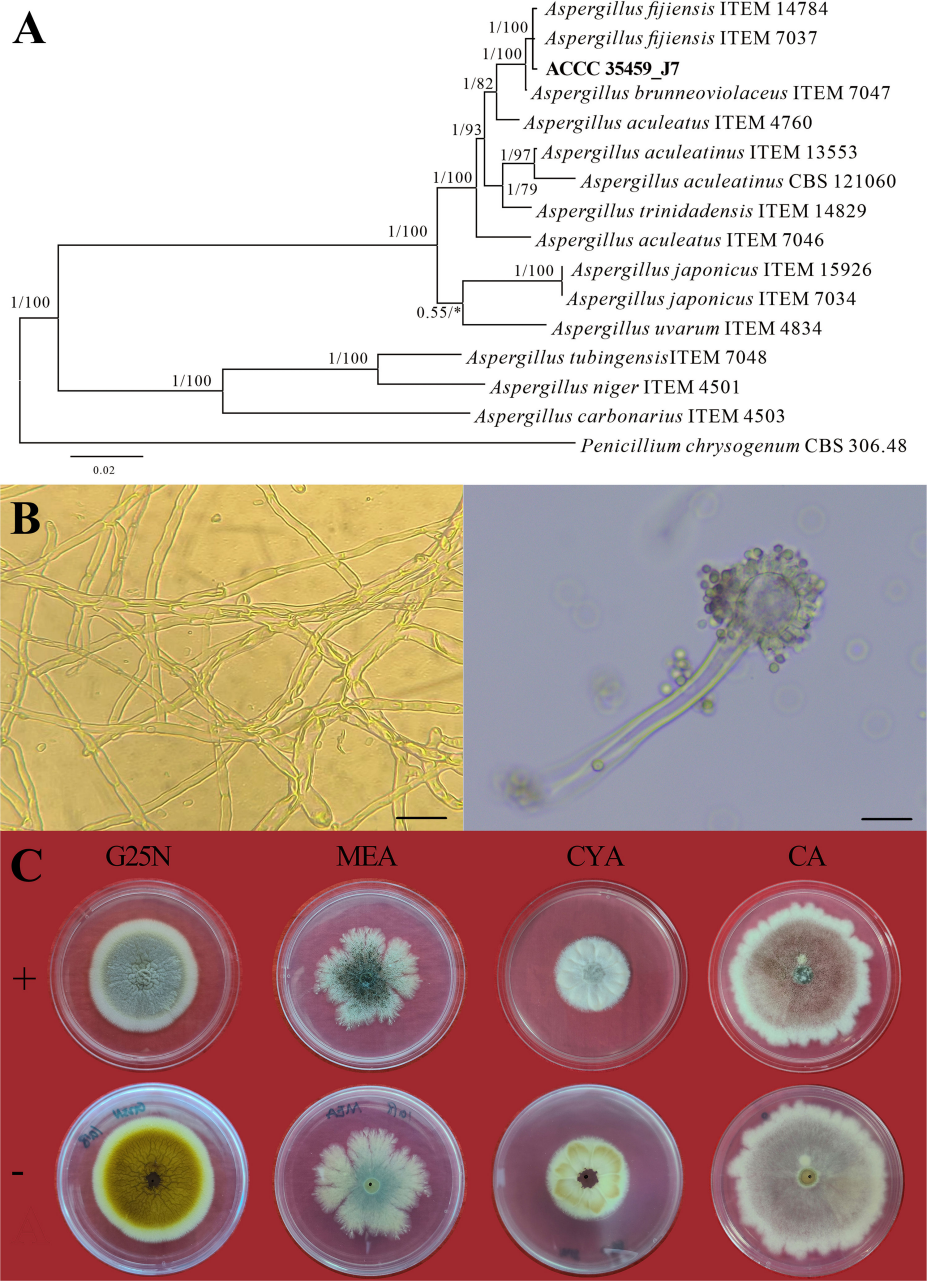
(A) Molecular phylogenetic analysis of *Aspergillus* and related taxa based on a combined ITS, *CaM*, *TEF-1a*, and *RPB2* sequences. Bayesian posterior probabilities (BPPs) and Bootstrap support values for Maximum Parsimony (MP) are displayed above or below the respective branches (BI/MP). The isolated strain J7 is in bold. (B) Microscopic characteristics of J7 hyphae and spores (40 × 10, scale bar = 20 µm). (C) Growth characteristics of strain J7 after 7 days of incubation on identification medium. +, front; −, back.

In the phylogram, sequences of J7 formed a well-supported (1/100, BI/MP) distinct clade with the *A. fijiensis*. Strain J7 is described as *A. fijiensis* J7 (Fig. 1A). The genetic markers used in phylogenetic analysis of strain J7, including *CaM*/ITS/*TEF-1a*/*RPB2*, are detailed in online resources. The conidial head of J7 was spherical and uniseriate, conidia ellipsoidal to slightly fusiform, brown. (Fig. 1B). The morphological characteristics of strain J7 across four types of media were as follows: *G25N medium*: colonies measured 60–65 mm in diameter, with neat, thick, and dense margins. Conidiophores were velvety and grayish-yellow on the front side, brown or bright yellow on the reverse. The colonies had concentric ring patterns, no exudate, and lacked soluble pigments. *MEA medium*: colonies were 35–40 mm in diameter, with irregularly cleft, thick edges. The conidiophore was velvety, adaxially white or greenish on the adaxial side, light yellowish-green on the reverse, with no exudate or soluble pigment. *CYA medium*: colonies grew to 40–45 mm in diameter, with neat, thick, and discernible edges. The basal mycelium had thick grooves and furrowed, and the conidiophores were velvet, white on the adaxial side, and white or yellow on reverse, lacking exudate and soluble pigmentation (Fig. 1C).

### Initial exploration of the pathogen antagonism of strain J7

**Fig 2:**
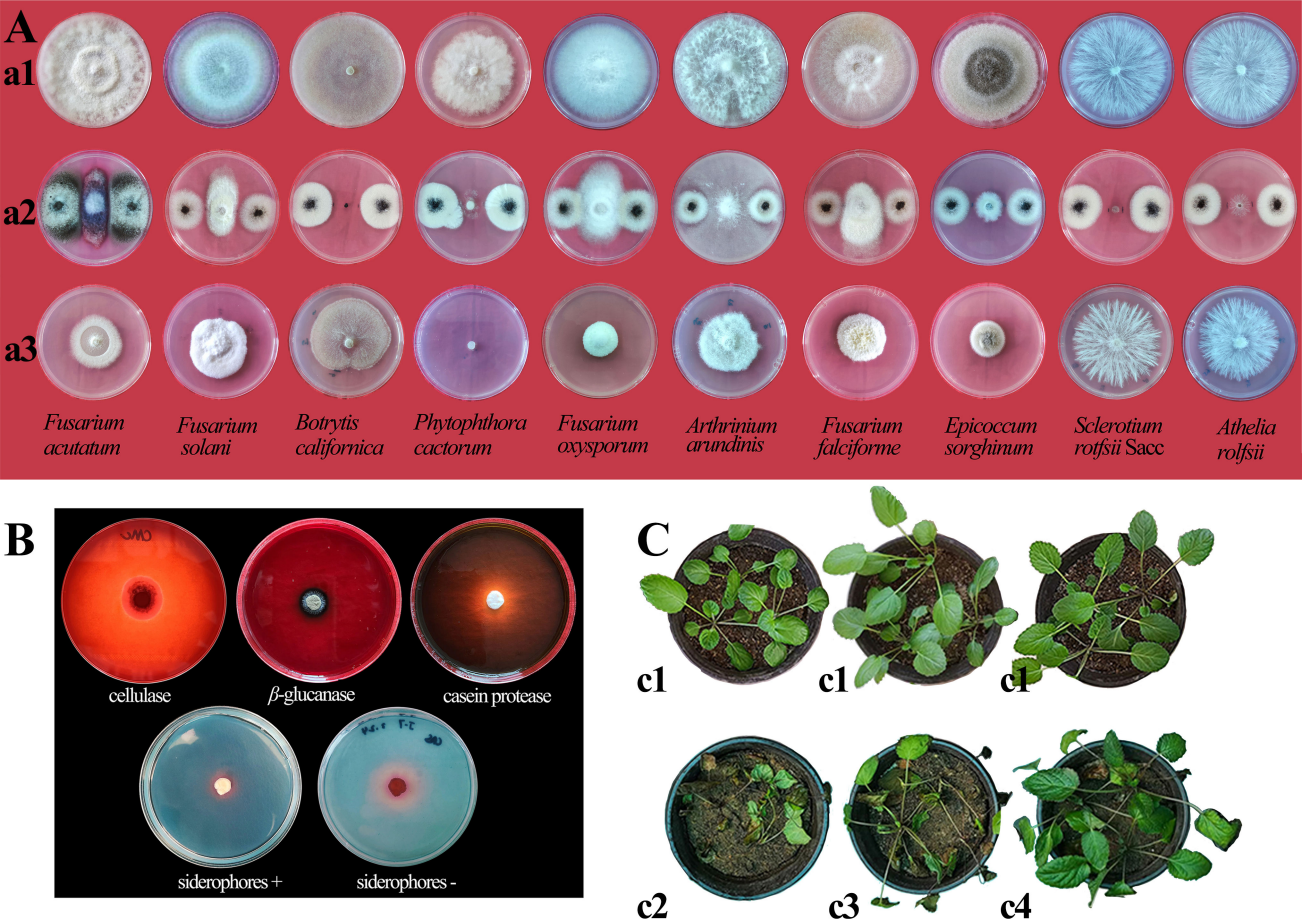
(A) Inhibitory effect of strain J7 on 10 pathogenic fungi, a1: control; a2: co-cultivation; a3: filtrate-modified medium culture. (B) Determination of strain J7’s ability to secrete cellulase, *β*-glucanase, casein protease, and siderophore. (C) Experimental effects of strain J7 on the biocontrol of *S. miltiorrhiza* blight in pots: c1: pre-experiment plant growth of each group; c2: control group; c3: treatment with 72% cymoxanil plus mancozeb (500 × dilution); and c4: J7 group treated with spore suspension. Number of independent experiments (ni. ex.) = 9; three plant/treatment.

In confrontation experiments, J7 inhibited 10 plant pathogens by over 50% (Fig. 2A-a2). After re-screening with 20% filtrate-modified medium, J7 produced inhibitory substances and effectively inhibited 10 plant pathogens (Fig. 2A-a3). After the above two screenings, strain J7 had the strongest inhibitory effect on *P. cactorum* with inhibition rates of 73.3% and 100.0%, respectively. Detection of extracellular enzymes secreted by J7 showed that it could secrete cellulase, *β*-glucanase, and casein protease (Fig. 2B), explaining the mechanism by which strain J7 could strongly inhibit the plant pathogens, as demonstrated by the formation of inhibition zones in co-culture assays.

### *In vivo* control of *S. miltiorrhiza* blight caused by *P. cactorum* using *S. miltiorrhiza* potted plants

Positive controls (72% cymoxanil plus mancozeb at a 500 × dilution) completely inhibited the growth of *P. cactorum in vitro* experiments, as expected (Online Resource Fig. S3). *S. miltiorrhiza* plants were pretreated with fungicide (cymoxanil plus mancozeb) or J7 spore suspension at the four- to six-leaf stage (Fig. 2C), resulting in a disease incidence reduction of 7.41% (*P* > 0.05) and 14.82% (*P* < 0.05), respectively. Correspondingly, the DI decreased by 11.53% (*P* > 0.05) for the fungicide and 23.05% (*P* < 0.05) for J7 (Table 1). These results indicate that strain J7 significantly reduced disease incidence and increased plant protection by 13.87% compared to the marketed fungicide, making it more effective in disease control.

**TABLE 1:** Efficacy of J7 in controlling blight on *S. miltiorrhiza* seedlings^*a*^

Treatment	Incidence rate (%)	DI (%)	CE (%)
Negative control group	96.30 ± 6.41	83.13 ± 10.65	–^*b*^
Positive control group	88.89 ± 11.11	71.60 ± 18.68	13.86
J7 (1 × 10^6^ spores/mL)	**81.48** ± 6.41	**60.08** ± 4.67	27.73

^*a*^ Negative control group, 3 mL of a 0.05% Tween-80 blank solution rooted; positive control group, 3 mL of 72% cymoxanil plus mancozeb (500 × dilution with 0.05% [vol/vol] Tween-80) was rooted; J7 (1 × 10^6^ spores/mL), 3 mL of J7 spore suspension was rooted. Analyses were performed using one-way ANOVA (independent-samples *t*-test). Bold values in a column indicate significant differences from negative controls (*P* < 0.05). Results are expressed as mean ± SD.

^*b*^ “–” indicates that this data is not meaningful.

### Growth-promoting effects of J7 in pots

**Fig 3:**
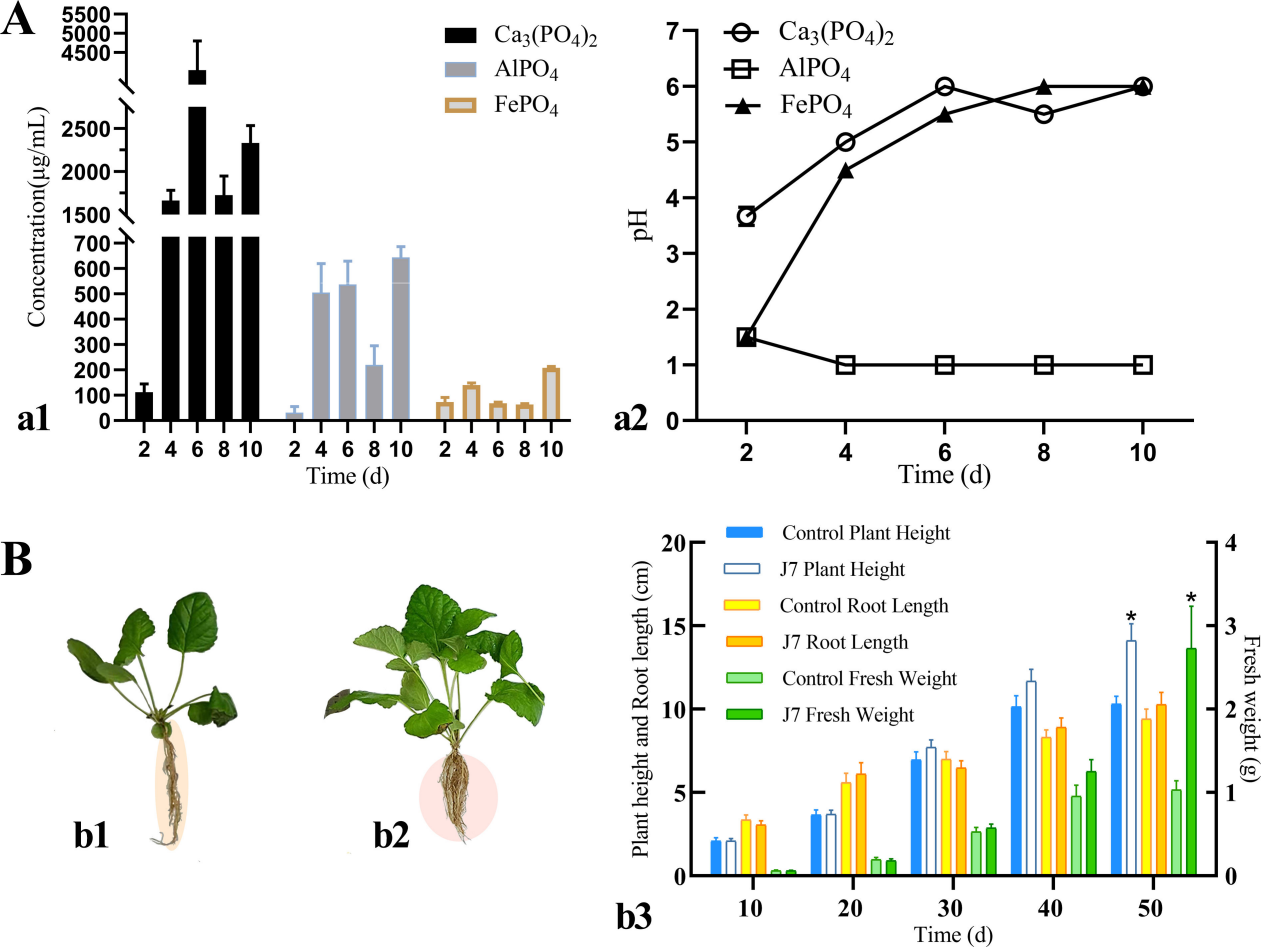
(A) Determination of J7’s ability to solubilize insoluble phosphorus (**a1**), the pH variation of the liquid medium during solubilization (**a2**). (B) Differences in the morphology of potted *S. miltiorrhiza* seedlings 50 days after the induction of J7 (**b2**) compared to the control group (**b1**); and the variation in biomass (**b3**), with the number of independent experiments (ni. ex.) = 5; three plant/treatment. Analyses were performed using one-way ANOVA (independent-samples *t*-test), *P < 0.05.

The results of the tests indicate that J7 may promote plant growth by solubilizing unavailable phosphorus in the soil and regulating soil’s pH. The solubility of J7 for inorganic phosphorus varied with pH. The solubility of J7 for Ca_3_(PO_4_)_2_ reached a peak value of 327.57 µg/mL on the 6th day, the solubility for AlPO_4_ reached a peak value of 64.36 µg/mL on the 10th day, and the solubility for FePO_4_ reached a peak value of 14.00 µg/mL on the 4th day (Fig. 3A-a1, a2). J7 could produce siderophores and IAA. After 7 days, colonies appeared on the CAS-PDA medium, and an orange-red reaction was observed around the fungal plug (Fig. 2B). The mixed group of fungal reagents exhibited a distinct color reaction, showing an orange-yellow bias with a red tint (Online Resource Fig. S4).

Root irrigation of *S. miltiorrhiza* seedlings at the four- to six-leaf stage with a spore suspension of strain J7 resulted in increased plant height, root length, and fresh weight. The *S. miltiorrhiza* of the experimental group showed morphological advantages, including thicker main roots and more abundant fibrous roots compared to the controls (Fig. 3B-b1, b2). During the first 40 days of cultivation, the spore suspension treatment showed a gradual but not significant growth-promoting effect on the seedlings. However, by day 50, significant differences emerged: fresh weight and plant height increased significantly to 2.39-fold (*P* < 0.05) and 1.37-fold (*P* < 0.05) of control values, respectively. While root length showed a 1.09-fold increase in the treatment group, this difference did not reach statistical significance (Fig. 3 b3).

### Growth-promoting and secondary metabolite-regulating effects of J7 inoculation in field experiments

In the first month, there was almost no difference in fresh weight between the experimental group and the control group. However, by the second month, the J7 treatment group began to show slightly higher fresh weight compared to the control group, although the differences in weight gain were not significant (Table 2, *P* > 0.05).

**TABLE 2:** Three consecutive months of weight gain in fresh weight of *S. miltiorrhiza* in field^*a*^

Treatment	Weight gained on 7/3 (g)	Weight gained on 7/4 (g)	Weight gained on 7/5 (g)
J7	1.07 ± 0.55a	9.00 ± 5.13b	30.81 ± 10.08c
Control	1.07 ± 0.62a	7.52 ± 3.82b	26.33 ± 9.99c

^*a*^ Results are expressed as mean ± SD. Analysis was carried out by one-way ANOVA (Student-Newman-Keuls multiple comparison test). Any two samples in a row with a common letter are not significantly different (*P* > 0.05).

**Fig 4:**
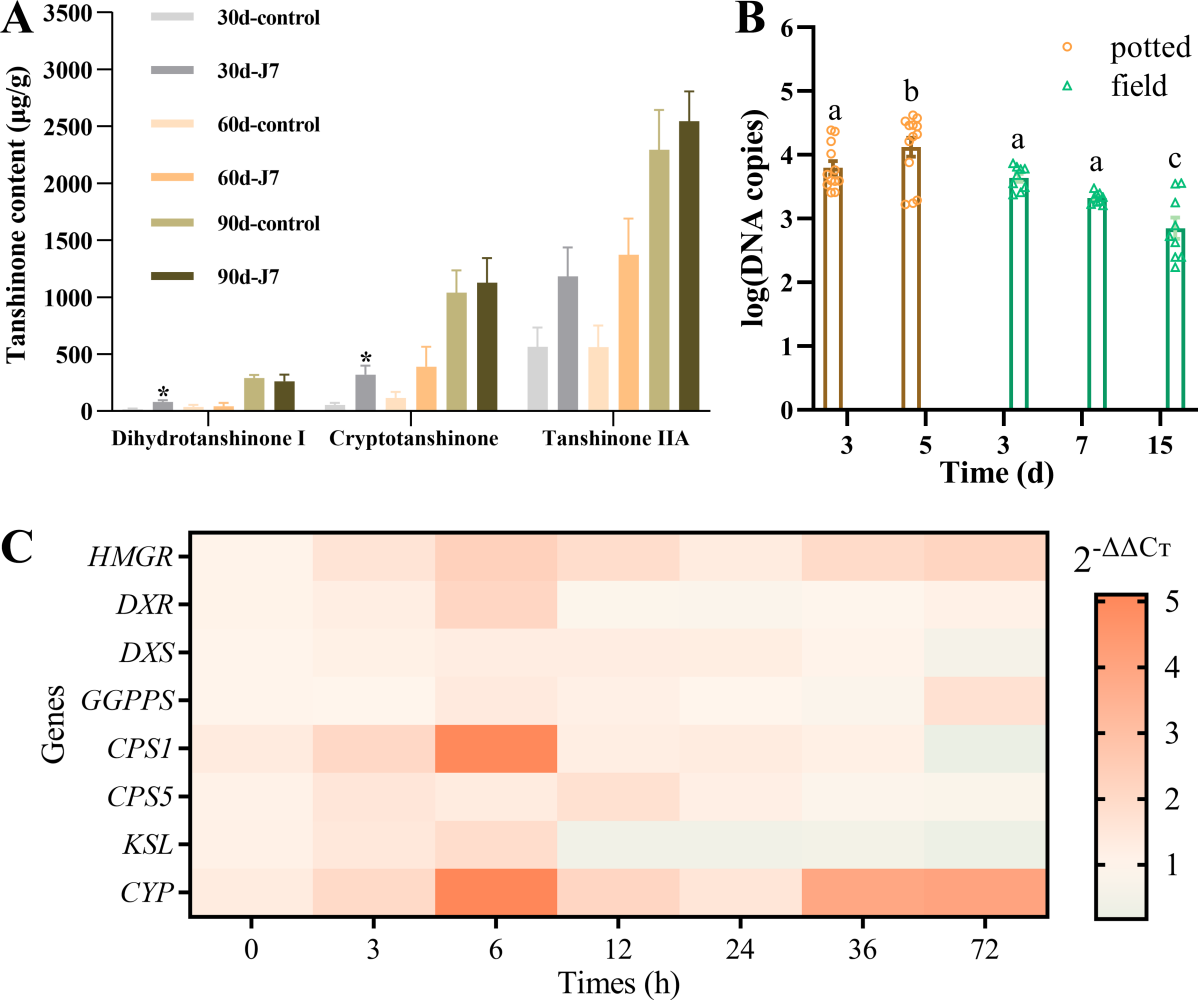
(A) Changes of tanshinone content in roots and rhizomes of *S. miltiorrhiza* in the field, number of independent experiments (ni. ex.) = 5; three plant/treatment. Analyses were performed using one-way ANOVA (independent-samples *t*-test), *P < 0.05. (B) Relative amount of fungal DNA copies was measured by qPCR of total genomic DNA extracted from the roots of *S. miltiorrhiza* grown in sterilized soil and the field, 3–15 days after irrigation with the suspension of transformant strain J7. Number of independent experiments = 5; three plant/treatment. Data are expressed as DNA copies of target DNA per 100 mg of plant. Different letters indicate statistically significant differences according to one-way ANOVA followed by the Student-Newman-Keuls multiple comparison test (*P* < 0.05). (C) Variation in the relative expression level of tanshinone synthesis-related genes 0, 3, 6, 12, 24, 36, and 72 h after the induction of strain J7: target mRNA extracted from roots of *S. miltiorrhiza* seedlings was determined by qPCR. Data are expressed as 2^−ΔΔC*t*^ values of target mRNA per 100 mg of plant based on the endogenous gene *β*-actin.

Quantitative analysis by HPLC showed that dihydrotanshinone I, cryptotanshinone, and tanshinone IIA in *S. miltiorrhiza* plants treated with J7 spore suspension exhibited an increase in the following three months. In the first month, the increases in dihydrotanshinone I and cryptotanshinone were significant (*P* < 0.05), with maximum increases of 4.64-fold, 7.49-fold, and 2.84-fold for dihydrotanshinone I, cryptotanshinone, and tanshinone IIA, respectively (Fig. 4A). Similar studies have shown that other fungi can significantly promote the biosynthesis and accumulation of tanshinones, particularly tanshinone IIA, in the roots of *S. miltiorrhiza*. For example, inoculation with the endophytic fungus *Mucor circinelloides* DF20 for 56 days significantly increased tanshinone IIA accumulation, reaching levels 22 times higher than the control (39). While DF20 showed a greater promotion of secondary metabolite accumulation compared to strain J7, this effect may be attributed to the experimental conditions of Chen et al. using sterile *S. miltiorrhiza* seedlings in a sterile system, which likely enhanced microbial stimulation under optimized conditions. Furthermore, their study did not evaluate the potential biocontrol properties of DF20.

### Colonization effects of J7 on *S. miltiorrhiza* roots

To quantify the colonization level of strain J7 in root tissues, we performed qPCR analysis targeting the *GFP* fragment carried by the strain. The Ct values obtained were converted to gene copy numbers using a standard curve (Online Resource Fig. S5) generated from serial dilutions of the target DNA fragment. This showed that colonization was maintained from 3 to 5 days after root inoculation with a suspension of 1 × 10^6^ spores/mL in the roots of the four- to six-leaf stage *S. miltiorrhiza* seedlings grown in pots with sterile soil, and the colonization level significantly increased with time, reaching approximately 1.41 × 10^4^ copies on day 5 (*P* < 0.05). The density of colonization in field-grown roots showed a gradual decrease over time. Compared to the initial 7 days of period after inoculation, a significant reduction (*P* < 0.05) in colonization was observed by day 15 (Fig. 4B). This progressive decrease in root colonization over the 15 days of observation period may be attributed to the complex field environment. Notably, strain J7 maintained a stable and effective colonization of *S. miltiorrhiza* roots throughout these two experiments, providing an important basis for subsequent *in vivo* experiments.

### Expression of tanshinone-related genes in the roots and rhizomes of *S. miltiorrhiza*

*Aspergillus* sp. J7 spore suspension treatment elicited transcriptional upregulation of tanshinone biosynthetic genes in *S. miltiorrhiza* seedlings. During the initial 0–6 h, all eight key enzyme genes exhibited synchronized upregulation: the mevalonate (MVA) pathway gene *HMGR* and methylerythritol phosphate (MEP) pathway gene *DXR* showed twofold induction compared to untreated controls, while downstream genes *CPS1* and *CYP76AH1* demonstrated fivefold activation, indicating prioritized diterpene skeleton assembly and oxidative modification. Beyond 6 h, transcriptional attenuation occurred in most genes except *CPS5*, with *DXR* and *KSL* entering sustained suppression. Notably, a secondary transcriptional resurgence emerged at 72 h, restoring expression of upstream regulators (*HMGR* and *DXR*) and downstream enzymes (*GGPPS* and *CYP76AH1*), while *HMGR* and *CYP76AH1* maintained persistently high expression throughout the observation period. Mechanistically, early-phase tanshinone synthesis relied on compartmentalized precursor fluxes—*HMGR*-driven cytosolic IPP (MVA pathway) and *DXR*-mediated plastidial IPP (MEP pathway)—coupled with *CPS1-CYP76AH1*-catalysed cyclization-oxidation cascades.

## DISCUSSION

Strain J7 was identified as *A. fijiensis*, and the taxonomic status of this species within the genus *Aspergillus* requires broader discussion and recognition. Currently, there are very few reports related to *A. fijiensis*, and it has not been included in the latest taxonomic and recognized species list by the International Commission on *Penicillium* and *Aspergillus* (https://www.aspergilluspenicillium.org/). The morphology of *A. fijiensis* is characterized by uniseriate conidial heads, in agreement with the results of the present study and those of Varga et al. (24). However, based on phylogenetic analyses, the production of an aspergillum-like conidial head does not guarantee that a given species belongs to *Aspergillus,* nor does it definitively identify which specific species of *Aspergillus* it might belong to (22, 24). It has been reported that the *β*-fructofuranosidase from *A. fijiensis* has been widely used in the commercial production of short-chain fructooligosaccharides (40). However, the species was initially archived as *A. niger*, it was reclassified as *A. japonicus* after 2015 and later reclassified as *A. fijiensis* (40). As shown in the phylogenetic tree presented in this paper, *A. fijiensis* exhibits molecular characteristics that are more closely related to *Aspergillus brunneoviolaceus*, yet *A. fijiensis* forms a well-supported (1/100, BI/MP) unique clade. The present study provides a more detailed delineation of the phylogenetic status of *A. fijiensis* through phylogenetic analyses, which supports the formal inclusion of *A. fijiensis* in the taxonomic and recognized species list of *Aspergillus* genera.

Based on the colorimetric reaction observed in CAS-PDA assays, J7 demonstrated potential siderophore production. Siderophores (500–1,500 Da) are microbial iron-chelators characterized by functional groups (catecholate, hydroxamate, carboxylate, or citrate) that coordinate Fe³^+^ via hard Lewis acid-base interactions (34, 41). Notably, *Aspergillus* species have been reported to produce a variety of metabolites with iron (III)-affinity functional groups, such as sphaeropsidin A, kojic acid, mellein, or neoaspergillic acid. These compounds are not only potential iron carrier molecules, but most also possess antimicrobial activity, such as the compound sphaeropsidin A, which controlled tomato late blight caused by *Phytophthora infestans* (KACC 48738) by up to 96% (42–44). These research advances in *Aspergillus* metabolite profiling provide crucial structural references for the siderophores produced by strain J7, while offering new perspectives for elucidating the molecular basis of its potent inhibitory activity.

Remarkably, under co-culture conditions, J7 demonstrated 73% inhibition of *P. cactorum* mycelial growth. This inhibitory effect significantly surpassed the 35% optimal inhibition rate observed in *A. flavipes* against *Phytophthora* strains (*P. parasitica* strains 05-287, 12-1, and *P. arecae*) (11). J7 culture filtrates demonstrated comparable efficacy to *Aspergillus montenegroi* SFC20200425-M27 in controlling *Phytophthora* pathogens. Specifically, J7 achieved 100% control against *P. cactorum*, matching the inhibition rate of *A. montenegroi* against *P. infestans*, which underscores J7’s potential as a high-quality biological control agent (42). Overall, in the genus *Aspergillus*, strain J7 was found to be more effective in controlling *Phytophthora* and was also among the top controllers of the pathogenic *P. cactorum*. Some of the more effective strains currently used to control plant infections of *P. cactorum* are *Pseudomonas fluorescens*, *Trichoderma harzianum*-1, *Enterobacter aerogenes*-2, and *Penicillium oxalicum* CX-1 (30, 45–48).

In *in vivo* experiments, strain J7 showed strong colonization ability in the roots and maintained its efficacy for over 3 months in field conditions. Plants inoculated with strain J7 demonstrated advantages in disease resistance, biomass accumulation, and secondary metabolite synthesis, with no observed phytotoxicity. However, the post-harvest lemon decay attributed to *A. fijiensis* by Thambi et al. (49) requires further verification, as their fungal classification was based solely on ITS sequencing. Additional validation using other molecular markers (e.g. CaM gene sequences) is required to confirm species identification. These limitations of the available studies, coupled with the paucity of reports on *A. fijiensis*, have led to inconsistent functional characterization of the species. In addition, the ability of strain J7 to control *P. cactorum* exceeded that of a commercial synthetic fungicide (cymoxanil plus mancozeb), and since *P. cactorum*-induced *S. miltiorrhiza* blight leads to root and leaf rot, root protection is an important part of *P. cactorum* control. In this study, J7 spore suspension was used for root irrigation to ensure a localized, high concentration of infection to achieve rapid colonization and protection by J7 in the root system of *S. miltiorrhiza* seedlings. Root infiltration is a quick and easy method to use compared to foliar spraying, which can lead to rapid volatilization and spore inactivation, or soil dressing, which can cause root damage to already stabilized seedlings. According to the J7 colonization results, the level of colonization increased significantly after 5 days of root irrigation and remained stable for more than 15 days in the field’s natural environment, further demonstrating that the application of the J7 not only has the advantage of environmental protection, but can also provide a more stable protective barrier for a longer period of time compared to commercial synthetic fungicides.

In conclusion, this study is the first to report the use of *A. fijiensis* for biological control of pathogenic fungi, which not only fills the gap of the current biocontrol in controlling the oomycete *P. cactorum*, but also promotes the growth of *S. miltiorrhiza* and regulates the accumulation of secondary metabolites. However, due to the complex metabolites of the *Aspergillus* genus, comprehensive screening of the complex metabolites of *Aspergillus* strains is essential to manage fungal and oomycete pathogens effectively, particularly in controlling mycotoxins, to ensure that biocontrol strains do not adversely affect human health and the environment. Current research suggests that species related to *A. aculeatus*, such as *A. fijiensis*, do not produce ochratoxins A and B, fumonisins, and aflatoxin B1 but rather a range of combinations such as asperparalines and okaramins (24). According to the results of Yan et al., *A. fijiensis* GDIZM-1 has also been reported to the control of the Asian citrus psyllid *Diaphorina citri* Kuwayama (50). Therefore, *A. fijiensis* has great potential to be developed as a new biocontrol agent.

## SUPPLEMENTAL MATERIAL

Supplemental material spectrum.03094-24-s0001.docx Additional experimental methods and results, including the formulation of the medium used in the experiment, sequence information on the phylogeny of strain J7, the standard curve needed for the statistics of the experimental data.

ASM does not own the copyrights to Supplemental Material that may be linked to, or accessed through, an article. The authors have granted ASM a non-exclusive, world-wide license to publish the Supplemental Material files. Please contact the corresponding author directly for reuse.
