# Supplementary material for: Endophytic Aspergillus fijiensis fungus J7 prevents disease and promotes growth in Salvia miltiorrhiza
Source: Microbiol Spectr. 2025 Jul 30;13(9):e03094-24. doi: 10.1128/spectrum.03094-24 (PMC12403878; doi:10.1128/spectrum.03094-24)
Supplement: Supplemental material — Additional experimental methods and results, including the formulation of the medium used in the experiment, sequence information on the phylogeny of strain J7, the standard curve needed for the statistics of the experimental data. [file spectrum.03094-24-s0001.docx]

**Endophytic *Aspergillus fijiensis*** **fungus J7 prevents disease and promotes growth in *Salvia miltiorrhiza***

**Running title:** *Aspergillus* sp. J7 stops plant disease and promotes growth

**Table S1. Composition of various media.**

| Media | [Formula](javascript:;) |
| --- | --- |
| Czapek ager (CA) | NaNO_3_ 3 g, K_2_HPO_4_ 1 g, KCl 0.5 g, MgSO_4_·7H_2_O 0.5 g, FeSO_4_·7H_2_O 0.01 g, sucrose 30 g, agar 15 g, [distilled](javascript:;) [water](javascript:;) 1000 ml |
| Czapek yeast extract agar (CYA) | K2HPO4 1 g, Czapek concentrate 10 ml, yeast extract 5 g, sucrose 30 g, agar 15 g, [distilled](javascript:;) [water](javascript:;) 1000 ml |
| Malt extract agar (MEA) | malt extract 20 g, peptone 1 g, glucose 20 g, agar 15 g, [distilled](javascript:;) [water](javascript:;) 1000 ml |
| 25% glycerol nitrate agar (G25N) | K_2_HPO_4_ 0.75 g, Czapek concentrate 7.5 ml, yeast extract 3.7 g, glycerol 250 g, agar 12 g, distilled water 750 ml |
| CMC | CMC 5 g, MgSO4·7H2O 0.1 g, (NH4)2SO4 0.5 g, K2HPO4 0.25 g, agar 18 g, distilled water 1000 ml |
| β-glucanase | β-glucan 3 g, NaNO_3_ 0.3 g, K_2_HPO_4_ 0.1 g, KCl 0.05 g, MgSO_4_ 0.05 g, FeSO_4_·7H_2_O 0.001 g, [Congo](javascript:;) [red](javascript:;) 0.004 g, agar 2 g, distilled water 100 ml |
| casein protease | [skim](javascript:;) [milk](javascript:;) [powder](javascript:;) 15 g, agar 20 g, distilled water 1000 ml |
| Organic /Inorganic phosphorus | glucose 10 g, MgSO_4_ 0.3 g, (NH_4_)_2_SO_4_ 0.5 g, NaCl 0.2 g, KCl 0.2 g, MnSO_4_ 0.03 g, FeSO_4_·7H_2_O 0.01 g, yeast extract 0.5 g, Ca_3_(PO_4_)_2_ 10 g/FePO_4_ 5g/AlPO_4_ 5g, distilled water 1000 ml |
| CAS-PDA | CAS-blue agar: A: 60.5 mg chrome azurol S in 50 ml distilled water+iron (III) solution 10 ml (1mM FeCl_3_﹒6H_2_O 0.027 g, 10 mM HCl 8.33 μl)+HDTMA 72.9 mg in distilled water 40 ml; B: distilled water 750 ml, agar 15 g, Pipes 30.24 g, 50 % NaOH solution (w/w) 12 g. 2 % PDA (w/w): potato 20 g, glucose 2 g, agar 15-20 g, distilled water 1000 ml |

**Methods for plotting phosphate standard curves**

Drawing the phosphate standard curve: suck 5 mg/L dipotassium hydrogen phosphate standard solution 0, 0.5, 1.0, 1.5, 2.0, 2.5, 3.0 mL into 10 mL volumetric flask, add 3 drops of dinitrophenol DNP indicator, the color of the solution becomes light yellow, then add 2. 5 mL of anti-colorant, shake the volumetric flask slowly to expel CO_2_, add RO water to 10 mL, after standing for 30 min, measure the OD_730_ _nm_ value with reference to the blank solution, and draw the standard curve using the OD value and the concentration of the standard solution (OD value is the vertical coordinate). After resting for 30 min, use the blank solution as a reference for zero adjustment, determine the OD_730 nm_ value, and draw the standard curve using the OD value and concentration of the standard solution.

**
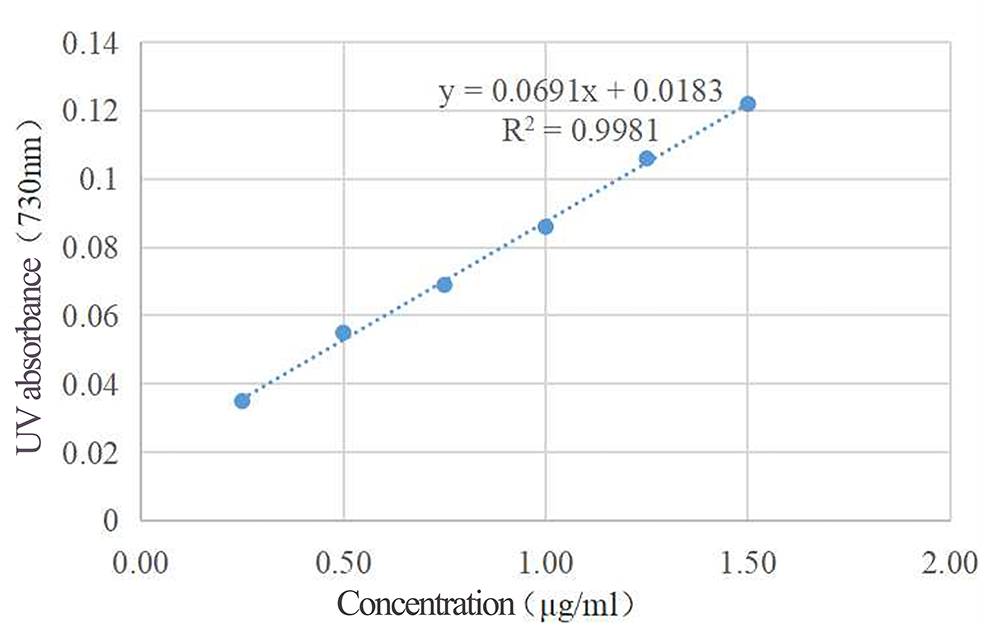
**

**Figure S1. Phosphate standard curve.**

**Chromatographic conditions**

Column: ZORBAX SB-C18 (3.0×100 mm, 1.8 μm); Column temperature: 35 ℃; Flow rate: 0.3 mL/min; Injection volume: 5 μL; Mobile phase: aqueous solution containing 0.1 % formic acid (A)-acetonitrile (B); A gradient elution program was used as follows: 39–39 % A (v/v) at 0–6 min, 39–10 % A (v/v) at 6–20 min, 10–39 % A (v/v) at 20–21 min, 39–39 % A (v/v) at 21–25 min; Detection wavelength: 270 nm; Validation data of the HPLC methodology are shown in the Supplementary Material.

**Methodological investigations for content determination**

Linearity, limit of detection and limit of quantification

The standards were weighed precisely, and 5 mL of methanol was added to make the reserve solution of dihydrotanshinone I, cryptotanshinone and tanshinone IIA with concentrations of 0.4200 mg/mL, 0.4000 mg/mL and 0.3800 mg/mL, respectively. The stock solution was diluted into different concentrations by gradient dilution, and 5 μL of the stock solution was injected into the liquid chromatograph and the peak area was recorded. Linear regression was performed using the least squares method to obtain the regression equation and linear range. The lowest limit of detection (LOD) and limit of quantification (LOQ) were taken as the injection concentration when the peak area was 3 times and 10 times the noise (S/N=3, 10), respectively.

Examination of precision

Intra-day precision: take the control solution into the sample 6 times in one day, and calculate the RSD value of the concentration of the 3 chemical components respectively; inter-day precision: take the control solution into the sample for 3 consecutive days, and the sample was injected 5 times per day, calculate the RSD value of the concentration of the 3 chemical components respectively.

Stability test

The test solution was injected into the sample at 0 h, 2 h, 4 h, 6 h, 8 h, 12 h, 24 h, 48 h, and 72 h. The concentrations of the three tanshinones in the sample were calculated and the RSD values were calculated respectively.

Repeatability

Six samples of Danshen were prepared and analyzed according to the same chromatographic conditions, the concentrations of the three tanshinones in the samples were determined and the RSD values were calculated.

Sample recovery

6 samples of *Salvia miltiorrhiza* root powder with known content were weighed precisely, and 0.075 g of each sample was added with the appropriate amount of 3 kinds of tanshinones control products to prepare a mixed solution, and the sample recovery and RSD values were calculated after analysis.

Results of Methodological Examination

Linear regression equation, standard curve, limit of detection, and limit of quantification.

The results of the regression equation and standard curve are shown in Fig. S2, and the linear ranges, detection limits, and quantification limits of the three components to be tested are shown in Table S2, which indicated that there was a good linear relationship between the concentration of the control product and the peak area (R^2^>0.999).


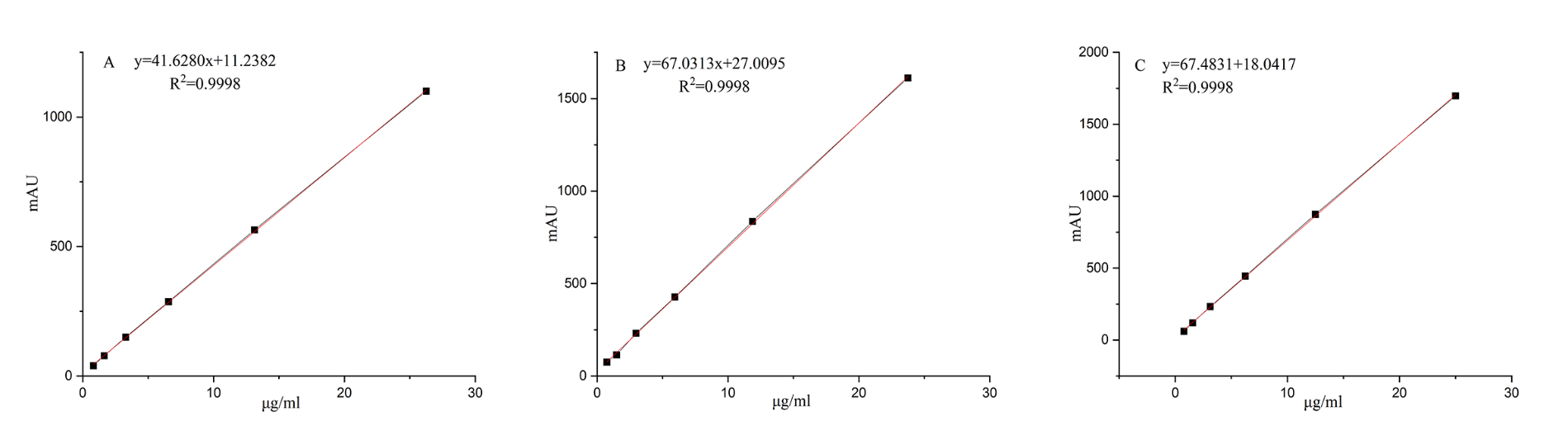


**Figure S2. Standard curve of tanshinone control. A: Dihydrotanshinone I; B: Cryptotanshinone; C: Tanshinone II.**

**Table S2. Linear range, limit of detection and limit of quantification of three tanshinones.**

| Components | linear range (μg/ml) | Limit of detection (μg/ml) | limit of quantification(μg/ml) |
| --- | --- | --- | --- |
| Dihydrotanshinone I | 0.82-26.25 | 0.4101 | 0.8203 |
| Cryptotanshinone | 0.74-23.75 | 0.1855 | 0.7422 |
| Tanshinone IIA | 0.78-25.00 | 0.1953 | 0.7813 |

Precision, stability, and reproducibility investigation

In the precision, stability, and reproducibility study, the precision RSD values of the three components were <5 %, indicating good precision of the instrument; the RSD of the three components was <5 % in 72 h, indicating good stability of the samples; and the RSD of the three components was <5 % in the reproducibility study, indicating good reproducibility of the method, see Table S3.

**Table S3.** **Precision, stability and repeatability of the three tanshinones examined.**

| Components | Intraday precision RSD (%) | Daytime precision RSD (%) | stability RSD  (%) | repeatability RSD  (%) |
| --- | --- | --- | --- | --- |
| Dihydrotanshinone I | 2.17 % | 2.16 % | 2.34 % | 1.34 % |
| Cryptotanshinone | 1.70 % | 1.54 % | 3.79 % | 1.20 % |
| Tanshinone IIA | 2.03 % | 1.73 % | 2.68 % | 1.15 % |

Sample recovery test

The results of the spiked recoveries are shown in Table S4. The average recoveries of the three components were between 95 % and 105 % with RSD <5 %, indicating that the spiked recoveries were good and the method was accurate and reliable.

**Table S4. Results of sample recovery experiments.**

| Components | Mean (%) | RSD (%) |
| --- | --- | --- |
| Dihydrotanshinone I | 102.80 | 1.24 |
| Cryptotanshinone | 99.31 | 1.08 |
| Tanshinone IIA | 98.92 | 1.10 |

**The genetic markers used in phylogenetic analysis of strain J7**

***> CaM***

ATTAGTCATCGGGCGGACACTCAAGCAGGGAATGTGTGAAACATACAGTCAATACGGCCGTCACCATCCTGGTCCGCCTCACGGATCATCTCATCGACTTCGTCATCGGTGAGCTTCTCGCCGATAGAGGTCATGACGTGGCGCAGCTCCGCGGCAGAGATGAAGCCGTTGTTATCGCGATCGAAGACCTTGAAGGCCTCACGGATCTCCTCCTCAGAGTCGGTATCCTTCATCTTACGGGCCATCATGGTAAGGAATTCTGGGCAGCTGTCAATACTATGTCCGGAGCTCGATGCATGGCCATCACATACCGGGAAAGTCGATAGTGCCGTTGTTGTCAGCGTCAACCTCGTTGATCATGTCCTGGAGCTCAGACTCGGAGGGGTTCTGGCCGAGAGAGCGCATAACGGTACCCAACTCCTTGGTGGTAATTTGTCCTGTAGCATGGATTCTGTCAGAAATGCGATCGATTCTTGATAGTTTCTGTGGCGTTTGCGGTATGGTGGCTATAGTGCCAGGTGATGCCAGGCGGGTTTCGTTGAGGGGTAAAGAACTACACTAACCATCGCCATCCTTGTCCTATCAGGGATCACGAGAAACGAATCAGTAAACATTGTTTTATAGCAGACGAGAAAGCATCGGGGTCGCTTACAAAAAGGGAGAAGCCCCCCCTT

**> ITS**

TTCCTCCGCCTTATTGATATGCTTAAGTTCAGCGGGTATCCCTACCTGATCCGAGGTCATCTGAGAAGATTGGGGGTCGAGGCAAGCCCCGGCCGGGCCCATAGAGCGGGTGACAGAGCCCCATACGCTCGAGGACCGGACGGTGCCGCCGTTTCTCTCGAGGCCCGCCCCCGGGGGGGCGCGGCCCAACAACCAGCGGGGCTGGAGGGGAGAAATGACGCTCGGACAGGCATGCCCCCCGGAATACCAGGGGGCGCAATGTGCGTTCAAAGACTCGATGATTCACTGAATTCTGCAATTCACATTAGTTATCGCATTTCGCTGCGTTCTTCATCGATGCCGGAACCAAGAGATCCATTGTTGAAAGTTTTGACTGATTGGTATCAATCGACTCAGACTGCACGCTTTCAGACAGTGTTCCATTGGGGTCTCCGGCGGGCGCGGTCCCGGGGGCAGGCCCCGGGCCGCCCGAAGGCGGGCCCGCCGAAGCAACAGGGTACGGTAAGCACGGGTGGGAGGTTGGGCCCCGAAGGACCCAGCACTCGGTAATGATCCTTCCGCAGGTTCCCCTACGGA

**> *TEF-1a***

GAATCCCCCGCACGTTACCACGGCGAACCTCcTTGACGGAAACGTTCTTGACGTTGAAACCAACGTTGTCACCGGGAACACCCTCCTTGAGCTGCTGGTGGTGCATCTCAACGGACTTGACTTCAGTGGTGACGTTGGCGGGAGCGAAGGTCACGACCATACCAGGGGTGATGACACCGGTCTCGACACGACCAACGGGCACAGTTCCAATACCGGAGATCTTGTAGACATCCTGGAGAGGAAGACGGAGAGGCTTGTTGGAGGGACGGACGGGAGGCTCAATGGCGTCGATGGCCTCGAGAAGGGTCTTACCGGTGGCCTTGCCGGACTTGGTCTCCTTCTCCCAACCCTTGTACCAGGGGCAGTTGGAGGAAACCTCGAGCATGTTGTCACCGTTGAAACCGGAGATGGGGACGAAGGGAACGGCCTTGGGGTTGTAGCCGACCTTCTTGATGAAGTTGGAGGTCTCCTTGACGATCTCGTTGTAACGGTCCTCGGACCACTTGCAGGTGTCCATCTTGTTGAGGGCAACAATGAGCTGACGGACACCGAGGGTGAAAGCGAGCAGAGCGTGCTCACGGGTCTGGCCATCCTTGGAGATACCAGCCTCGAATTCACCAGTACCGGAGGCAATGATGAGGATAGCACAATCGGCCTGGGAGGTACCAGTGATCATGTTCTTGATGAAGTCACGGTGACCGGGGGCATCTAAGGGGAATGGGTTAGATGATGGATGATAGCTTGTAGAGTCATGGGAGATAACTTACCAATGACGGTAACCTCATACTTGGCAGTCTGGAACTTCCAGAGAGCGATATCGATGGTGATACCACGCTCACGCTCGGACTTGAGCTTGTCAAGACCCACGTTCCTT

***> RPB2***

GGACTGGTTGTGATCTGGGAAGGGAATGATACTGGCACAAACACCAAGGATCATACTGGGATGGATCTCGCAGTGAGTCCAAGTGTGCGCCTTTTGACTCAGAATGGAGCGTACACGCTTGTTCGGATCGTGTAGTTCCTCATCCGGAAGAGCATAACCAGCCTGGAGTTGCTTGGAAATTTCGAGATCTTCCGGAGTCATGGAAATCATGATGGTCTCTTCTTCTTCAGCATCGACGTACTCCACTACACCAGACTTCACCAGGCCATCCCAACCAAAGTAACGATCGCGGCGGTCTTCTGGATCCAAGTCTGGAGGCAGGTCTTTATCTTGTTCTAGTTTGCGGATGTGTTCTTTGTTGAGCACCAGAGAGCCGCAGTTTTCACTCTTCGGGTCATTGTCAATGACGTAGAGCGGTCGGCAGACACGTCCAGCATCGGTGAAAATCTTGAACTCCCGCTCACGAATGTCTCGAATCAGACTGACCTCGTGCGAAATCATGTTGCGGCGACGAAGGGAAAGCATCGTGTTGACAAGGTGAGCGGGGTCTCTGTGGATACCAACCCAGACACCGTTGACAAAAACCTTGGTAGCGTTCGGTGTCACTTGAGGTTCGAACTCCTCGAGGACTTCCATATTACGCTGAATCATGAAATCAATGATAGGCTCGCTGGGCGTACCGACAGTGATGTAGCACATGAGAGCCAAGTTCTTGACCAAACCACAAGCTTGACCTTCAGGGGTTTCAGCCGGACACACCAGGCCCCAATGAGTGTTGTGAAGCTGACGAGGCTTGGCGATCTTTCCGTCTCGCCCGATGGGTGTATTGGTTCGGCGAAGATGGGACAAGGTGGAGGCGTAAGTGTAACGACTGAGCACTTGAGACACACCGGCCTTGGAGCTAGCTGCCTTCTTCTGCTCGCCCCAGTTACCCGTAGCAAGAGCATACTTCAATCCTCCCGTCAAGGTGCTAGCCTTGATACCAATGTTCAGATAAATCTCTCTGTTCGTCTCCACGCACCGCTGAACATACCGCTGTAGATCGCGGGTGACGCGGGTGAACAAGACTCGGAAAAGGTCGCAAGAAGAGACCGCA

**
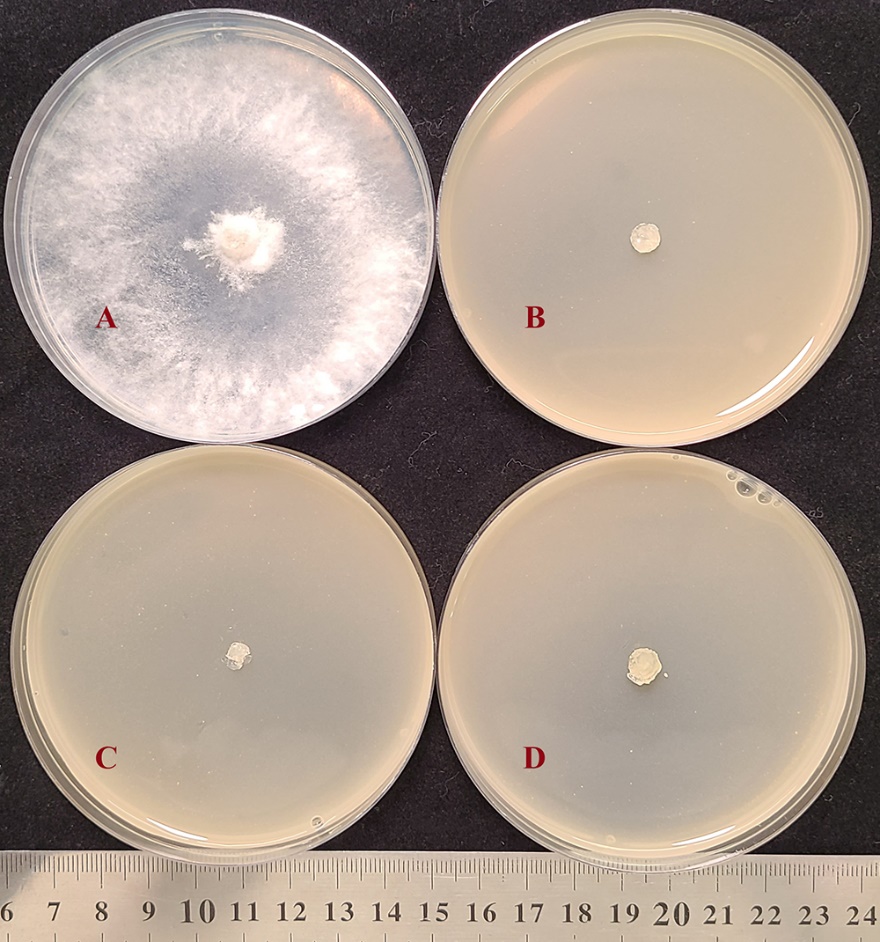
**

**Figure S3. Inhibition of *P. cactorum* by 72 % cymoxanil plus mancozeb (500×dilution) after 7 days of dark incubation at 25 ℃. Three biological replicates were used for each treatment group. the blank group was inoculated in PDA medium and the experimental group was inoculated in PDA medium supplemented with 72% cymoxanil plus mancozeb (500×dilution). A:** **Blank group; B, C, D: Experimental group.**

**
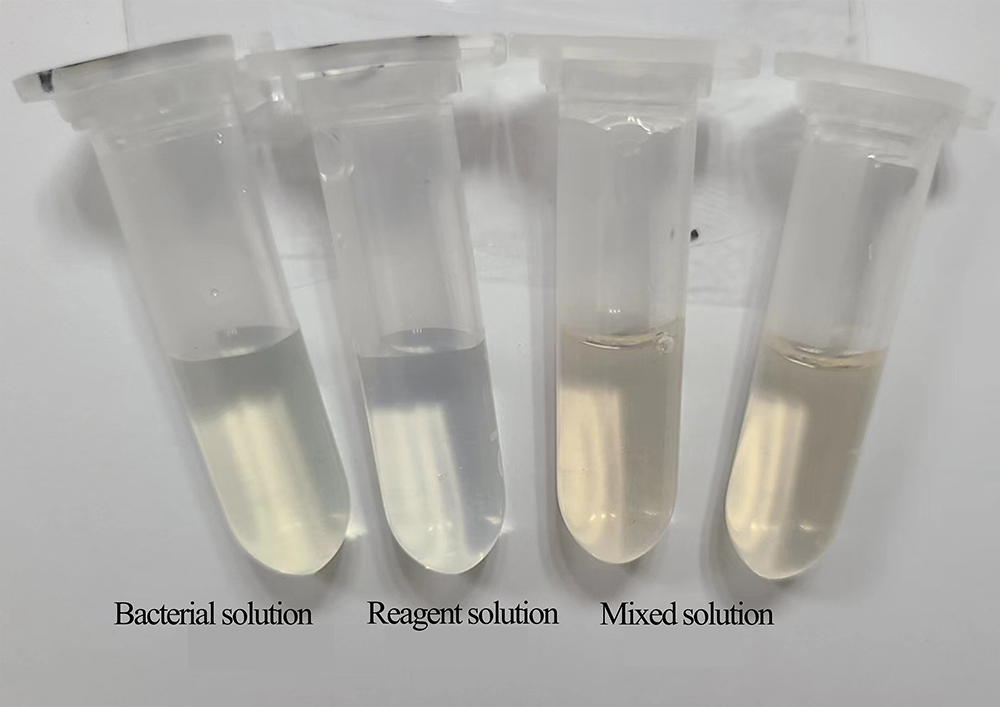
**

**Figure S4. Colour reaction of strain J7 with Salkowski's reagent.**

**
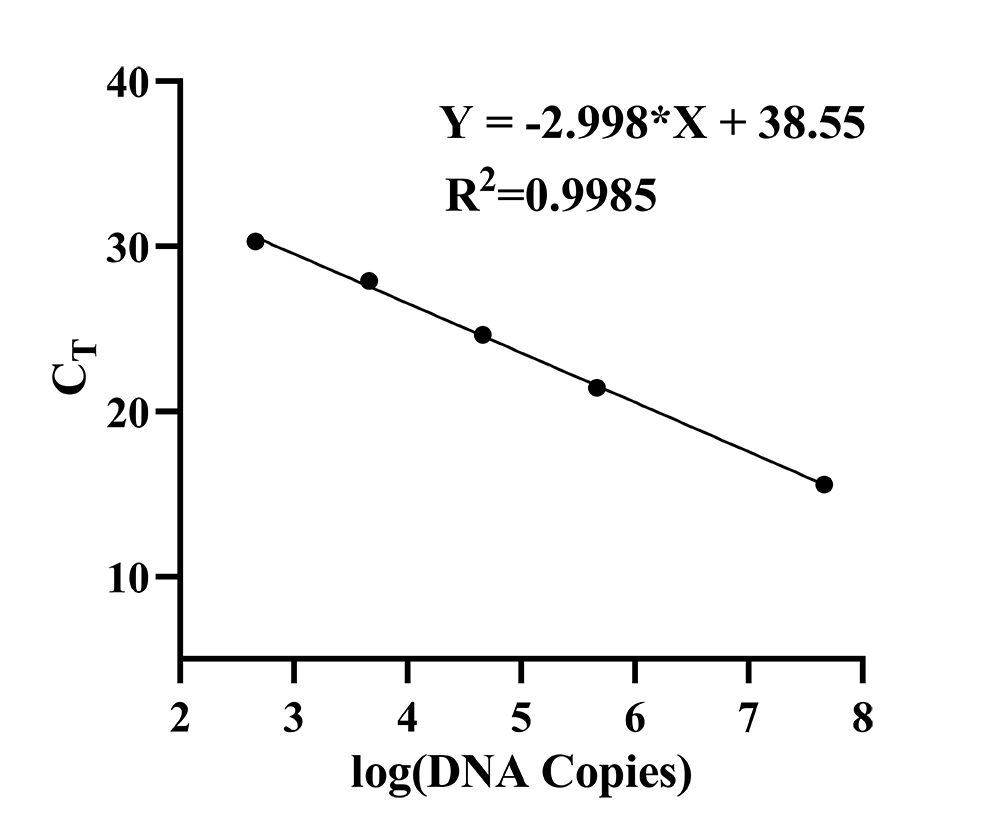
Figure S5. Standard curves obtained by plotting the logarithm of known DNA copies (10-fold dilution series from 10 pg to 10-4 pg/10 μL reaction) of the plasmid (pCAMBIA1303-TrpC-Hygro-gpdA-GFP) against the Ct values.**
